# The Book World of Medicine and Science

**Published:** 1894-04-28

**Authors:** 


### The Book World of Medicine and Science.

Characteristics. By S. Weir Mitchell. (The Century Company, New York.)

"This book is a broken record of portions of the lives of certain friends of mine." So the author prefaces his first chapter. It is a book without a plot, deficient in incident, and the *dramatis personæ* are common-place individuals who invariably act just as any other reasonable-minded persons would under similar circumstances. Herein, probably, lies the charm of the book, the freedom from extravagance and exaggeration, the simple and unadorned delineation of rather colourless lives unfolds a panorama of by no means unattractive pictures, and is an exhibition of considerable literary skill. Although lacking the brilliance, the sparkle, and the overwhelming genius of Guy de Maupassant, the wit, the humour, and the philosophy of Oliver Wendle Holmes, the author is evidently a worshipper at the shrine of these artists; his description of pain and its alleviation by morphia is, perhaps, the most powerfully-drawn picture in these pages, and is suggestive of a distant reflection of Maupassant's account of an attack of neuralgia and its magic banishment by narcotics. The somewhat laboured epigrams, paradoxes, and *reductiones ad absurda* with which the pages of this book abounds, are faint echoes of the finer notes of Holmes. It is a thoughtful book for thoughtful readers.

A Short Guide to the Examination of Lying-in Women. By Professor Crede and Professor Leopold, translated by William Wilson, M.A., M.B. (Oxon), Radcliffe Travelling Fellow. (London: Henry Kimpton, 1894, pp. 48.)

This pamphlet is an extract from the authors' treatise on obstetrics for midwives issued by order of the government of Saxony, containing the two chapters dealing with the external examination of patients and with the method of disinfection and cleansing. There can be but little doubt that the diagnosis of the position of the child by external examination of the mother is a proceeding which is very much neglected in England, while it is one which, if carefully practised, may do more than many other much more troublesome procedures to prevent the occurrence of puerperal diseases. Patients who are not examined may die of accident or mal-position but they tend but little to die from fever, and it clearly would be a great gain if a definite conception of the position of the child and the progress of the labour could be gained without introducing the finger into the vagina. The object of this little book is to show how this can, to a large extent, be done, and the conclusion is arrived at that in the majority of cases of normal labour a single vaginal examination will suffice, and that if after this the method of external examination be constantly used no repetition of it will be required.

